# Learning musculoskeletal anatomy through new technologies: a randomized clinical trial*

**DOI:** 10.1590/1518-8345.3237.3281

**Published:** 2020-08-12

**Authors:** Elena Sonsoles Rodríguez-López, Sofía Olivia Calvo-Moreno, Eduardo Cimadevilla Fernández-Pola, Tomás Fernández-Rodríguez, Jesús Guodemar-Pérez, Montserrat Ruiz-López

**Affiliations:** 1 Camilo José Cela University, Madrid, Spain.

**Keywords:** Anatomy, Competency-Based Education, Teaching, Innovation, Ultrasonography, Anatomy, Regional, Anatomia, Educação Baseada em Competências, Ensino, Inovação, Ultrassonografia, Anatomia Regional, Anatomía, Educación Basada en Competencias, Enseñanza, Innovación, Ultrasonografía, Anatomía Regional

## Abstract

**Objective:**

to investigate the influence of the application of new methodologies on learning and the motivation of students of the Anatomy discipline.

**Method:**

randomized, longitudinal, prospective, intervention study. Sixty-two students were recruited to assess the impact of different methodologies. The sample was randomized to compare the results of teaching with a 3D atlas, ultrasound and the traditional method. The parameters were assessed through a satisfaction evaluation questionnaire and anatomical charts. Repeated measures ANOVA was used to determine statistical significance.

**Results:**

in terms of the usefulness of the seminars, 98.1% of the students considered them to be very positive or positive, stating that they had stimulated their interest in anatomy. The students who learned with the 3D atlas improved their understanding of anatomy (p=0.040). In general, the students improved their grades by around 20%.

**Conclusion:**

the traditional method combined with new technologies increases the interest of students in human anatomy and enables them to acquire skills and competencies during the learning process.

## Abstract (pt)

**Objetivo:**

investigar a influência da aplicação de novas metodologias na aprendizagem e a motivação de alunos da disciplina de Anatomia.

**Método:**

estudo de intervenção, prospectivo, longitudinal e randomizado. Um total de 62 alunos foram recrutados para se avaliar o impacto de diferentes metodologias. Eles foram distribuídos de forma aleatória para comparar os resultados de ensino usando o atlas 3D, o ultrassom e o método tradicional. Os parâmetros foram medidos por meio de um questionário de avaliação de satisfação do aluno e uma prova usando lâminas anatômicas. O teste ANOVA de medidas repetidas foi usado para determinar significância estatística.

**Resultados:**

relativo à satisfação dos alunos, 98,1% consideraram muito positivo ou positivo o uso dos seminários, além de afirmarem que o seminário havia estimulado seu interesse pela anatomia. Os alunos submetidos ao método com o atlas 3D apresentaram uma melhora em sua compreensão de anatomia (p=0,040). De forma geral, os alunos melhoraram suas pontuações em aproximadamente 20%.

**Conclusão:**

o método tradicional, junto com as novas tecnologias, contribui para aumentar o interesse dos alunos pela Anatomia Humana assim como na aquisição de habilidades e competências em seu processo de aprendizagem.

## Abstract (es)

**Objetivo:**

investigar la influencia de la aplicación de nuevas metodologías en el aprendizaje y en la motivación de los alumnos en la asignatura Anatomia.

**Método:**

estudio de intervención, prospectivo, longitudinal y aleatorizado. Fueron convocados 62 alumnos para evaluar el impacto de diferentes metodologías. Se los dividió aleatoriamente para comparar resultados entre enseñanza con atlas 3D, ultrasonido y metodología tradicional. Los parámetros fueron analizados mediante un cuestionario de evaluación de satisfacción y láminas anatómicas. Se usó ANOVA de medidas repetidas para determinar la significancia estadística.

**Resultados:**

el 98,1% de los alumnos consideraron muy positiva o positiva la utilización de los seminarios, afirmaron asimismo que el seminario había estimulado su interés por la anatomía. Los alumnos que realizaron el aprendizaje con atlas 3D mejoraron su compresión de la anatomía (p=0,040). En general, los alumnos mejoraron sus puntajes en alrededor del 20%.

**Conclusión:**

el método tradicional con el agregado de las nuevas tecnologías permite a los alumnos incrementar su interés por la Anatomía Humana, así como adquirir habilidades y competencias en su proceso de aprendizaje.

## Introduction

Human anatomy courses are obligatory in every year of Health Sciences programs. Studying and understanding the subject requires different skills on the part of students, as well as considerable effort to consolidate their knowledge of the different body structures, communication through precise technical language and adequate body spatial orientation. There is a complex balance between knowledge acquisition, skills and learning results. It is challenging and necessary for professors to upgrade their skills in order to improve the quality of their teaching.

The learning process, combined with the challenge of memorizing relevant information, also involves the skill of using resources to find, assess and apply this information. However, the volume of content in the subject of anatomy leaves students little time to improve their understanding and integration of concepts^(^ 1 ^)^.

Lectures are effective for transmitting information and guiding study programs^(^ 2 ^-^ 3 ^)^, however, numerous studies have found that using different methodologies, such as problem-based teaching, practices and other types of participatory methodologies carried out in groups, strengthens the integration of knowledge acquired during lectures^(^ 4 ^)^. This is particularly relevant in subjects such as human anatomy, where textbooks reflect a different reality than the possibility of observing anatomical structures in real time through ultrasound^(^ 5 ^)^. Those who study anatomy with the help of imaging techniques develop a positive perception of the subject that is not only short-term but lasts for years^(^ 4 ^)^.

For this reason, the issue needs to be addressed in today’s university community, due to the growing demand of students who want to enhance their professional profiles in the clinical realm, with a curriculum increasingly oriented toward new technologies. To this end, a program was designed, comprised of different teaching methodologies in appropriate proportions, to maximize both knowledge acquisition and the development of skills. It also sought to develop a yardstick for measuring learning that assesses knowledge acquisition, combined with those competencies related to the subject. In line with the above, the present study was based on the hypothesis that educational interventions with new methodologies represent an effective strategy for enhancing knowledge of musculoskeletal anatomy. The purpose of this study was to investigate the influence of the application of new methodologies on learning and the motivation of students of the Anatomy discipline.

## Method

A randomized, longitudinal, prospective, intervention study was conducted. The study was implemented over a period of eight weeks. Sixty-two students (20 women and 42 men) in their first year of the Physiotherapy and/or Nursing program of Camilo José Cela University were recruited to assess the impact of an innovative methodological approach, constituting an experimental strategy. The students participated voluntarily and every candidate who expressed interest in being part of the study was included. They were invited to attend seminars, with each session lasting 90 minutes. The methodology involved comparing the results obtained from a study group that used a 3D atlas (n=23), another study group that used ultrasound (n=20) and a control group that received traditional lectures (n=19). The teaching staff was shared among the three groups. The groups were assigned according to the group of practices to which each student belonged, and the methodology selected for each group was randomly decided by the teaching staff. The randomization process was done through a random numbers table generated by the software Epi Info version 7.1.4. None of the students were familiar with the teaching methodology that would be applied to them.

The control group was administered the content according to a traditional lecture-based methodology, using anatomy textbooks for the different regions and their planes, in addition to palpation. In the 3D atlas study group, a mixed methodology was applied, composed of lectures and a 3D atlas, and the palpation activity followed the seven-step method^(^ 6 ^)^. As for the ultrasound study group, a mixed methodology was also applied, with lectures and ultrasound, and palpation practices were carried out with ultrasound. The topics and number of hours for each group were the same.

To assess the objective, different parameters were collected:

*Sociodemographic data:* age, sex, previous university studies, number of hours *per* week dedicated to the study of anatomy, usual anatomy study method and if the student had any prior knowledge of anatomy.

*Satisfaction evaluation questionnaire:* upon completing the methodology seminars, they were given a Likert-type scale questionnaire with five rating options (1- Very positive, 2- Positive, 3- Normal, 4- Negative and 5- Very negative), in order to assess the subjective perception of the study methodology.

*Anatomy charts:* Prior to the seminar and immediately after, the students’ learning was assessed using anatomy charts selected by the teaching staff (shoulder region, cross section of the arm, anterior region of the abdomen, lateral compartment of the leg and internal region of the ankle). Each chart was graded on a score of ten and an average was calculated for the six charts assessed.

The study was conducted in accordance with the ethical standards of the Declaration of Helsinki^(^ 7 ^)^, and the patients’ data was kept confidential^(^ 8 ^)^. Before participating, the students received written information on the study objectives and procedures and agreed to participate by signing an informed consent form. This study was appraised by the Research Ethics Committee of Camilo José Cela University (Spain, ENMDEAP). It was registered in the Australian and New Zealand Clinical Trials registry, under Registration No. ID378931. Afterwards, the 25 elements of the checklist were verified according to the CONSORT Statement^(^ 9 ^)^.

The statistical analysis was performed with the program SPSS 22.0 (SPSS Science, Chicago, United States). A descriptive study was performed on each of the variables in tables with means ± SD (standard deviation) and a confidence interval of 95%; the nominal variables were expressed in percentages. The Shapiro-Wilk test indicated normal distribution of the quantitative variables (p>0.05), as well as homogeneous distribution among the different study groups (p>0.05). Repeated measures analysis of variance (ANOVA) with a linear model with Bonferroni adjustment was used to test the profile of the change in pre- and post-seminar results, of the three study groups and the pairwise comparison according to time and group. A confidence interval of 95% and level of significance of p<0.05 were established, which is universally considered adequate in biomedical research.

## Results

**Figure 1:**
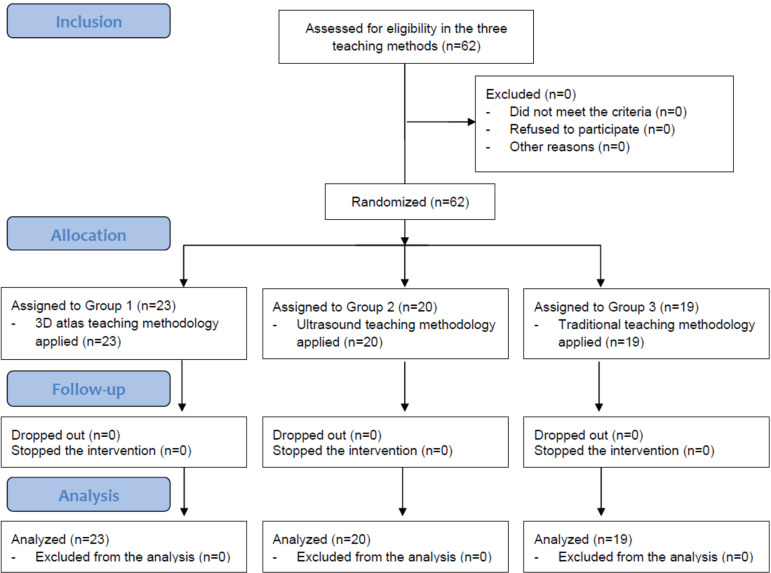
Flowchart of the study. Madrid, Spain, 2018

Sixty-two students aged 25.63 ± 7.62 years, 32.3% women and 67.7% men, participated in the study conducted from February to April 2018 (Figure 1). 41/1% had no prior university studies, 27.9% were graduates, 18% had graduated in Physical Activity and Sports Sciences, 1.6% in Nursing, 6.6% in Occupational Therapy and 3.3% in Speech Therapy and 1.6% were physicians. At the time of the study, 56.7% were not exercising any profession. Concerning the usual anatomy study method, 53.3% chose a combination of textbooks, videos and 3D atlas for their studies, whereas 33.3 % only used textbooks, dedicating 3.03 ± 3 hours of study per week to anatomy. 70% reported having prior knowledge of anatomy.

Regarding the data obtained for the primary objective, the mean score of the students for the charts before the seminars was 3.46 ± 1.8 points out of ten, the highest rate of correct answers was in relation to the lateral compartment of the leg chart (4.74 ± 3.12 points) and the highest rate of incorrect answers occurred in the lateral compartment of the leg and internal region of the ankle, with 0.50 ± 1.55 points. After 90 minutes of seminars with a different methodology (Table 1), the students improved their scores by around 20% in the completion of the charts (15.53% in the 3D atlas group, 10.03% in the ultrasound group and 28.98% in the traditional teaching group). In the data from the repeated measures ANOVA, there was significant interaction among the group and time factors in the Abdomen-1 group (*F* [2.59]= 6.42; p=0.003; n^2^= 0.179). The post-hoc analysis, in particular, revealed statistically significant differences between the results before and after the seminar in the ultrasound group (p=0.049) and traditional teaching group (p=0.009). For the ankle chart, there was no significant interaction between the group and time factors (*F* [2.59]= 0.129; p=0.879, n^2^= 0.179), but there was a relevant effect of time on the type of teaching (*F* [1.59]= 8.61; p=0.005; n^2^= 0.127); significant improvement was noted in the post-hoc analysis in the ultrasound group (p=0.044). Neither was there any significant interaction between the group and time factors (*F* [2.59]= 0.930; p=0.879, n^2^= 0.145) for the arm chart, but time did have a strong effect on the type of teaching (*F* [1.59]= 4.74; p=0.033; n^2^= 0.074). For the shoulder, abdomen-2 and leg charts, there were no significant changes (p>0.05).

**Table 1:** Analysis of covariance for completion of the anatomy charts. Madrid, Spain, 2018

		3D atlas group (n=23)		Ultrasound group (n=20)		Control group (n=19)		p-value^‡^
		Mean (SD*)	p-value^†^	Mean (SD*)	p-value^†^	Mean (SD*)	p-value^†^	
Shoulder	Pre	4.43 (3.18)	0.871	4.60 (3.05)	0.858	3.21 (3.83)	0.880	0.611
	Post	4.30 (3.81)		4.95 (4.95)		4.26 (3.69)		
Arm	Pre	1.22 (3.23)	0.274	3.15 (4.23)	0.130	1.84 (3.84)	0.265	0.930
	Post	2.39 (3.95)		4.90 (4.49)		3.16 (4.15)		
Abdomen1	Pre	1.70 (1.42)	0.202	1.60 (1.87)	0.049	2.05 (1.61)	0.009	0.003
	Post	2.17 (1.46)		2.40 (1.78)		3.16 (1.86)		
Abdomen2	Pre	4.26 (2.98)	0.961	5.50 (3.44)	0.072	4.53 (2.93)	0.915	0.300
	Post	4.30 (3.86)		3.75 (3.65)		4.63 (3.71)		
Leg	Pre	5.04 (2.45)	0.683	4.40 (3.05)	0.560	4.84 (2.79)	0.822	0.757
	Post	5.30 (3.54)		4.00 (3.94)		5.00 (3.09)		
Ankle	Pre	0.57 (2.15)	0.147	0.60 (1.23)	0.044	0.32 (0.94)	0.127	0.879
	Post	1.57 (2.84)		2.10 (4.66)		1.47 (2.50)		
Total	Pre	2.93 (1.83)	0.541	3.46 (1.69)	0.875	2.84 (1.91)	0.164	0.658
	Post	3.25 (2.39)		3.55 (3.26)		3.64 (2.31)		

*SD = Standard deviation; ^†^p-value (time) = Pairwise comparison results (based on adjustment for multiple comparisons: Bonferroni); ^‡^p-value (time*group) = Within-subject test results (based on sphericity assumption)

Regarding the secondary objective of the study, measured through the satisfaction evaluation questionnaire (Table 2), 98.1% of the students rated the usefulness of the seminar as very positive (67.9%) or positive (30.2%). The same percentage claimed that the seminar had stimulated their interest in anatomy. The interactive style of the seminar was scored as very positive (63.5%) or positive (32.7%) by the students. All the students felt that the seminar should be repeated for another anatomical region. There were no significant differences among the different groups in the assessment of the items from the satisfaction evaluation questionnaire (p>0.005), except for the question “Did the seminar improve your understanding of anatomy?” where there were significant differences between the groups (χ² [4]=10.05; p=0.040): 46.4% of the students who were taught through a 3D atlas, 17.9 % who used ultrasound and 35.7% who received traditional teaching scored this aspect as very positive.

**Table 2:** Results of the satisfaction evaluation questionnaire. Madrid, Spain, 2018

	Very positive	Positive	Normal
1. In general, was the seminar beneficial for you?	67.9 %	30.2 %	1.9 %
2. Did the seminar stimulate your interest in anatomy?	67.9 %	30.2 %	1.9 %
3. Did the seminar improve your understanding of anatomy?	52.8 %	41.5 %	5.7 %
4. Did you like the interactive style of the presentation?	63.5 %	32.7 %	3.8 %
5. Was it well organized?	53.8 %	40.4 %	5.8 %
6. Do you consider that this approach is useful for helping learn about the clinical relevance of anatomy?	66.0 %	34.0 %	0 %
7. Would you like to repeat this seminar for another anatomical region?	69.8 %	30.2 %	0 %

## Discussion

Students feel overwhelmed by the large volume of information they receive throughout the course^(^ 10 ^-^ 11 ^)^. Incorporating new technologies into anatomy classes enables viewing the system in vivo and improves students’ understanding of the spatial relationship and orientation among various anatomical structures^(^ 12 ^)^. In this study, the mean percentage of the scores in the satisfaction evaluation questionnaire indicated that the students considered the use of new technologies as highly advantageous in their learning process. Since the use of mixed methodologies, involving a combination of textbooks, online material and videos, among others, facilitates understanding and the acquisition of skills and competencies, it can be concluded that this approach deepens anatomical knowledge and its clinical application^(^ 13 ^-^ 14 ^)^.

The results of this study shed greater light on the question as to whether teaching with new technologies fosters and increases interest in human anatomy. The data from the present study, as in other studies^(^ 15 ^-^ 17 ^)^, suggests that the application of new technologies, such as 3D models, is useful and stimulates interest in anatomical learning; in relation to teaching based on 3D atlases, the students reported understanding human anatomy better. Interestingly, when students completed the charts of the different anatomical regions again, the rate of correct answers was higher when they received lectures combined with traditional atlases. However, the three methodologies boosted the students’ percentage of correct answers when completing the charts. These results suggest that students believe that a multimodal teaching method that uses lectures, a traditional atlas, a 3D atlas and ultrasound will have a positive impact on their ability to learn about anatomy.

Similar studies have been conducted with medical, nursing and podiatry students^(^ 18 ^,^ 19 ^)^. There was a greater diversity in the sample of the present study since, even though they were all physiotherapy and/or nursing students, around 60% had prior university studies. They were all first-year students and, based on previous studies^(^ 20 ^)^, the level of participation is greater in this population since the level of tiredness they experience is less than among those in upper-year courses.

Among the approaches utilized in the standard teaching of anatomy is the use of textbooks, lectures or clinical cases^(^ 20 ^)^, perceived by students as less effective methods. Other authors^(^ 5 ^)^ support this idea, concluding that only three of the textbooks examined provided a solid explanation and correct foundation for understanding anatomy. However, the use of some radiodiagnosis techniques, such as ultrasound, enable dynamic viewing of anatomical structures in real time, without the aggression that a dissection may entail or the static aspect of images from an anatomical atlas^(^ 21 ^)^, where it is possible to combine palpation and localization of the structures described, linking it to a process of perception mediated by touch and images, thereby avoiding possible errors in the discrimination of anatomical structures during palpation^(^ 9 ^,^ 22 ^)^.

It is for this reason that, for years, various studies have sought to demonstrate the usefulness of ultrasound as a supplementary method for studying anatomy, with promising results^(^ 23 ^-^ 28 ^)^, even though a recently published review concluded that further investigation is necessary in this regard^(^ 29 ^)^.

In relation to the influence of new technologies on the anatomical learning process, the students increased their number of correct answers by 20%, with results similar to other studies^(^ 13 ^)^, but differing from the findings of another study^(^ 19 ^)^, although it was concluded in the latter that technological improvement of the simulator used could be decisive for obtaining results similar to those in the present study. It should be noted that the highest rate of improvement occurred in the control group, which suggests that, despite the review in the aforementioned studies, the traditional method may be the most appropriate for enhancing the academic performance of students.

The limitations of the study were sample size, as well as the quantification of learning and short-term satisfaction. The authors feel that it would be interesting in future studies to increase the number of students, make comparisons with other traditional methods, as in the case of learning through cadavers, or verify whether the degree of satisfaction and the knowledge acquired are maintained over the years.

## Conclusion

The use of new technologies, as a support to traditional teaching methods in human anatomy courses, increases the interest of students and enables them to acquire skills and competencies in their learning process. The three teaching methodologies – lectures, 3D atlas and ultrasound – suggest a potentially beneficial effect on learning human anatomy, without finding any differences between them. This study emphasizes the importance of compiling students’ preferences in order to optimize the teaching methods used in human anatomy study plans.
